# Imaging and Analysis of Presynaptic Calcium Influx in Cultured Neurons Using synGCaMP6f

**DOI:** 10.3389/fnsyn.2019.00012

**Published:** 2019-04-16

**Authors:** Johannes Brockhaus, Bianca Brüggen, Markus Missler

**Affiliations:** Institute of Anatomy and Molecular Neurobiology, Westfälische Wilhelms-University, Münster, Germany

**Keywords:** synapse, live imaging, paired pulse facilitation, high voltage activated calcium channels, synaptic transmission, genetically encoded calcium indicator

## Abstract

Presynaptic Ca^2+^ influx through voltage-gated calcium channels (VGCCs) is a key step in synaptic transmission that links action potential (AP)-derived depolarization to vesicle release. However, investigation of presynaptic Ca^2+^ influx by patch clamp recordings is difficult due to the small size of the majority of synaptic boutons along thin axons that hamper clamp control. Genetically encoded calcium indicators (GECIs) in combination with live cell imaging provide an alternative method to study Ca^2+^ transients in individual presynaptic terminals. The indicator GCaMP6f was developed for fast speed and high sensitivity in detecting Ca^2+^ transients even in subcellular compartments. We fused GCaMP6f to synaptophysin (synGCaMP6f) to enrich the calcium indicator in presynaptic boutons of transfected primary hippocampal neurons to study presynaptic Ca^2+^ changes in response to individual APs or short bursts. Changes in fluorescence intensity were evaluated by normalization to control level or, alternatively, by normalization to maximal fluorescence using the calcium ionophore ionomycin. Measurements revealed robust Ca^2+^ transients with amplitudes that depend on parameters like the number of APs, stimulation frequency or external calcium concentration. Our findings indicate an appropriate sensitivity of synGCaMP6f for studying total presynaptic Ca^2+^ transients induced by single APs or short bursts that showed little rundown of the response after repeated bursts. Moreover, these recordings are fast enough to even study short-term plasticity like paired pulse facilitation (PPF) and frequency dependence of Ca^2+^ transients. In addition, synGCaMP6f could be used to dissect the contribution of different subtypes of VGCCs to presynaptic Ca^2+^ influx. Our results demonstrate that synGCaMP6f allows the reliable analysis of changes in presynaptic calcium concentration at many individual synaptic boutons in parallel and provides the possibility to study the regulation of this important step in synaptic transmission.

## Introduction

In presynaptic terminals, action potentials (APs) induce calcium transients that are the key factor in the translation of electric activity to transmitter release (Neher, 1998). Calcium transients depend on voltage-gated calcium channels (VGCCs) and their regulation, for example, *via* metabotropic receptors by retrograde action of released transmitters. Also other factors like the subunit composition or splice variants of the VGCCs (Hoppa et al., 2012; Thalhammer et al., 2017), the calcium storage within the endoplasmic reticulum (de Juan-Sanz et al., 2017), or other interacting molecules like neurexins (Brockhaus et al., 2018) shape the presynaptic calcium transient in response to invading APs and, by this, modulate the efficiency of the presynaptic release machinery. However, recording of presynaptic calcium currents is challenging due to the small size of normal presynaptic boutons. Direct recording with patch clamp pipettes is hardly possible due to size restrains and investigations of bigger presynapses like the squid giant axon (Augustine et al., 1991), the calyx of Held (Borst et al., 1995) or mossy fiber boutons (Li et al., 2007) are technically challenging and limited by concerns these specialized synapses allow generalized conclusions.

To study smaller, prototypical presynaptic boutons with respect to calcium dynamics, fluorescent indicators may provide a solution. Fluorescence recordings of changes in the concentration of free calcium ions were first performed with an organic fluorescent sensors like the widely used double-wavelength indicator Fura-2 (Tsien et al., 1985; Takahashi et al., 1999), or the single wavelength dyes Oregon-green-BAPTA (OGB), Fluo5F and others. These dyes were often applied as AM-esters (Tsien, 1981) or *via* the patch pipette solution for use in combination with electrophysiological recordings (Augustine, 1994). These dyes provided substantial insight into many intracellular processes and the central role of calcium.

However, the AM-ester loading does not differentiate between pre- and post-synaptic sites. Therefore sophisticated further labeling in combination with synaptic receptor blocking would be required to investigate pure presynaptic calcium transients (Kirischuk et al., 1999).

Also loading *via* a patch pipette is challenging (Ermolyuk et al., 2012; Liu et al., 2014) as labeling of presynaptic boutons by diffusion is slow along thin axons. Furthermore, diffusion is impeded by the small volume of boutons and low dye concentrations, which would restrain recordings to boutons near the soma.

More recently, the small-molecule synthetic indicators got competition from the growing class of genetically encoded calcium indicators (GECIs). GECIs usually combine a fluorescent protein, for example, a modified EGFP, with a calcium chelator protein like calmodulin that switches the fluorescence intensity after binding of Ca^2+^ ions. GECIs made it much easier to visualize intracellular calcium changes as they can be applied by transient transfection *in vitro* or *in vivo* several days before investigation (Lin and Schnitzer, 2016). Moreover, GECIs can be incorporated in the mouse genome, and allow targeting to specific cell types by use of adequate promotors. Currently, the arguably most popular GECI is GCaMP (Nakai et al., 2001; Tian et al., 2009) which combines a circular permutated green fluorescent protein (GFP) with calmodulin at the C-terminus and the calmodulin binding region of the chicken myosin light chain (M13) at the N-terminus. Calcium binding to the calmodulin initiates an interaction with the M13 leading to a conformation change that induces a substantial increase in the fluorescence of the GFP moiety. GCaMP underwent several improvements and version GCaMP6f is now available in three forms (slow, medium and fast), with slightly distinct properties (Chen et al., 2013; Horikawa, 2015). With GCaMP6f, the fast version of the GCaMP6 family, the dynamic range, i.e., the fluorescence increase factor from calcium-free to calcium-saturated, is now above 50 (Chen et al., 2013), and thus rivals the range of the small-molecule synthetic indicators. Originally intended to observe spontaneous activity in living animals, the GCaMP6 indicators may cause some problems when permanently expressed in transgenic models *in vivo*, inducing increased buffering in the targeted compartments (Singh et al., 2018) and aberrant electrical activity similar to interictal spikes (Steinmetz et al., 2017). Very recently, modifications of Cav1.3 calcium currents by GCaMP6f in transient transfection of HEK cells are described next to the *in vivo* problems and a GCaMP-X that claims to overcome these problems was introduced (Yang et al., 2018), but this is not tested here.

GCaMP was used in transient transfections of neurons for visualizing calcium dynamics in a wide range of experiments on subcellular compartments: in presynaptic compartments GCaMP3 elucidated the role of overexpressed α2δ subunits of VGCCs (Hoppa et al., 2012), GCaMP6f was used to study the role of presynaptic active zone plasticity (Glebov et al., 2017) and allowed to investigate the modulation of presynaptic calcium transients by neurexin (Brockhaus et al., 2018). Postsynaptically, it was used to investigate single synapse responses of NMDA receptors after coupling GCaMP6f to PSD95 (Reese and Kavalali, 2016). In addition, it was applied in an analysis of long-term plasticity of individual synapses in rat hippocampal brain slice culture (Wiegert et al., 2018).

Here, we show that GCaMP6f targeted to presynaptic terminals of primary hippocampal neurons can be used as a reliable indicator to elucidate important properties of the AP-induced presynaptic calcium transient such as dose dependence, short-term plasticity and contribution of the different subtypes of VGCCs.

## Materials and Methods

### Animals

Mice of either sex were used for neuronal cultures derived from timed-pregnant females at E17. Animal experiments were performed at the University of Münster in accordance with government regulations for animal welfare and approved by the Landesamt für Natur, Umwelt und Verbraucherschutz (LANUV, NRW, Germany), license numbers 84-02.05.20.11.209 and 84-02.04.2015.A423.

### Cell Culture

Dissociated primary neurons were prepared in Hank’s Balanced Salt Solution (HBSS) from hippocampi as described (Neupert et al., 2015). Briefly, cell suspensions obtained after 0.25% trypsin and trituration were plated onto 18 mm glass coverslips (Menzel-Glaeser, Braunschweig, Germany) coated with poly-L-lysine (Sigma) at a density of 55,000 cells/coverslip. After 4 h at 37°C in plating medium (MEM, 10% horse serum, 0.6% glucose, 1 mM sodium pyruvate), coverslips were inverted onto a 70%–80% confluent monolayer of astrocytes grown in 12-well plates (Falcon), and incubated in Neurobasal medium supplemented with B27, 0.5 mM glutamine and 12.5 μM glutamate. After 3 days, media were refreshed with Neurobasal medium supplemented with B27, 0.5 mM glutamine and 5 μM AraC. Cultures were maintained at 37°C in a humidified incubator with an atmosphere of 95% air and 5% CO_2_. Neurons were transfected at day-*in vitro* (DIV) 14 using lipofectamine (Thermo Fisher Scientific, Waltham, MA, USA), and experiments performed between DIV17 and DIV21.

### Expression Vectors

For Ca^2+^ imaging, we used GCaMP6f (Chen et al., 2013) or a version, that coupled GCaMP6f to synaptophysin and was driven by the synapsin promotor (synGCaMP6f) as described earlier (Brockhaus et al., 2018). For better identification of neuronal morphology, in some experiments, we used pMH4-SYN-tdimer2-RFP (RFP, T. Oertner, Hamburg, Germany).

### Immunohistochemistry

Primary hippocampal neurons were transfected with RFP and synGCamP6f at DIV 14 using lipofectamin, following the supplier’s protocol (Thermo Fischer Scientific). Three days after transfection, cells were washed in PBS containing 0.4% sucrose and fixed in 4% PFA containing 0.4% sucrose for 10 min. All the following washing steps were performed using PBS. After washing, cells were permeabilized using 0.3% PBS-Triton X-100 for 10 min and then blocked in PBS containing 5% normal goat serum (NGS) for 30 min. Primary antibodies [rabbit-anti-GFP, Abcam (ab290); rabbit-anti-GFP, Santa Cruz (sc8334); mouse-anti-synapsin 1, Synaptic Systems (106001)] were diluted 1:1,000 in PBS containing 5% NGS and cells were incubated overnight at 4°C. After several washing steps, secondary antibodies [goat-anti-rabbit IgG (H+L), Alexa Fluor 488, Thermo Fisher Scientific (A-11034); goat-anti-mouse IgG (H+L), Alexa Fluor 647, Thermo Fisher Scientific (A-21235)] were diluted 1:1,000 in the same way and applied for 1 h. After washing, cells were mounted with Dako Fluorescence Mounting Medium.

Alternatively, to analyze the localization of synGCaMP6f at active presynaptic terminals, we preincubated the neurons for 45 min with an oyster550-labeled antibody against the luminal domain of the vesicular GABA-transporter (VGAT, 1:200; Synaptic Systems, catalog #131103C3) as a marker for (inhibitory) presynaptic boutons (Neupert et al., 2015). The marker was visualized with an excitation wave length of 540 nm by use of a monochromator (Visitron Systems, Puchheim, Germany) and an emission filter of 562 nm. The labeling prior to the recording of calcium dynamics allowed to examine the co-localization of spots with stimulation-induced increase in the synGCaMP6f fluorescence.

### Image Acquisition and Analysis

For antibody staining, confocal images were acquired with a spinning disc Axio Observer-Z1 (Visitron) with an EMCCD camera (ImagEM 512 CCD, Hamamatsu) using a 40× immersion objective and lasers at 488 nm, 568 nm and 647 nm wavelength. Z-stacks were acquired with 0.2 μm intersection.

Image processing was performed using FIJI/ImageJ (National Institute of Health, USA). Confocal stacks from five subsequent slices were background subtracted (rolling ball radius = 20 pixels) and collapsed to a projection of average intensity. All images were adjusted for brightness and contrast for presentational purposes.

### Ca^2+^ Imaging

To determine presynaptic Ca^2+^ influx, primary neurons were transfected at DIV14 with synGCaMP6f (see above) and, as indicated, additional plasmids like RFP were co-transfected. Three to five days post transfection, neurons growing on glass coverslips were placed in a recording chamber mounted to an inverted microscope (Observer.A1, Zeiss, Oberkochen, Germany) and superfused at 1.0–1.5 ml/min with bath solution (temperature 32°C), containing (in mM): NaCl 145, KCl 3, MgCl_2_ 1.5, CaCl_2_ 1.5, glucose 11, HEPES 10; pH 7.3 adjusted with NaOH; to suppress postsynaptic signaling, 10 μM 6-cyano-7-nitroquinoxaline-2,3-dione (CNQX), 25 μM D, L-2-amino-5-phosphonovaleric acid (AP5), and 10 μM bicuculline were added. All chemicals were obtained from Sigma (St. Louis, MO, USA), except calcium channel blockers (Alomone Labs, Jerusalem, Israel). A stimulation electrode, built by two platinum wires of 10 mm length in 10 mm distance was positioned with a micromanipulator (MPC-200, Sutter Instrument, Novato, CA, USA) and neurons were stimulated with 50 Hz trains of 1, 3, 10 or 30 current pulses (1 ms, 55 mA). Ca^2+^ transients were visualized and recorded (10 or 20 ms exposure time, frame rate 100 or 50 Hz, binning 2: 0.46 μm per pixel) with a CMOS camera (Orca Flash4.0, Hamamatsu, Japan), a LED-light source (SpectraX, Lumencor, Beaverton, OR, USA) using the green channel (excitation at 470 ± 20 nm) or red channel (640 ± 20 nm) and controlled by VisiView software (Visitron Systems, Puchheim, Germany). As a standard, 20 frames were recorded before the stimulus train was triggered. For stimulation with one AP, four individual recordings with 10 s time interval were averaged frame by frame to improve the signal-to-noise ratio.

To normalize the change in fluorescence to the maximal fluorescence (F_max_), in a subset of experiments the Ca^2+^ ionophore ionomycin (10 μM) was applied after halting the perfusion at the end of the recording to saturate the Ca^2+^ indicator. For each cell, the maximum of the stimulation-induced Ca transients was compared to the maximal fluorescence, obtained with ionomycin, to calculate the relative fluorescence increase.

In some experiments, Ca^2+^ channel antagonists were added by direct application into the recording chamber. During halted perfusion, 10 μl of stock solution (ω-conotoxin GVIA, 200 μM; ω-agatoxin IVa, 40 μM; nifedipine, 2 mM; SNX-482, 50 μM) was applied to the bath solution (volume ≈1 ml) above the recording area at least 1 min before recording, leading to calculated final antagonist concentrations of 2 μM (ω-conotoxin), 400 nM (ω-agatoxin), 20 μM (nifedipine) and 0.5 μM (SNX-482), respectively. The antagonist concentration was proven to be sufficient by a full blockade after subsequent application of all four blockers.

### Data Analysis

**Figure 1:**
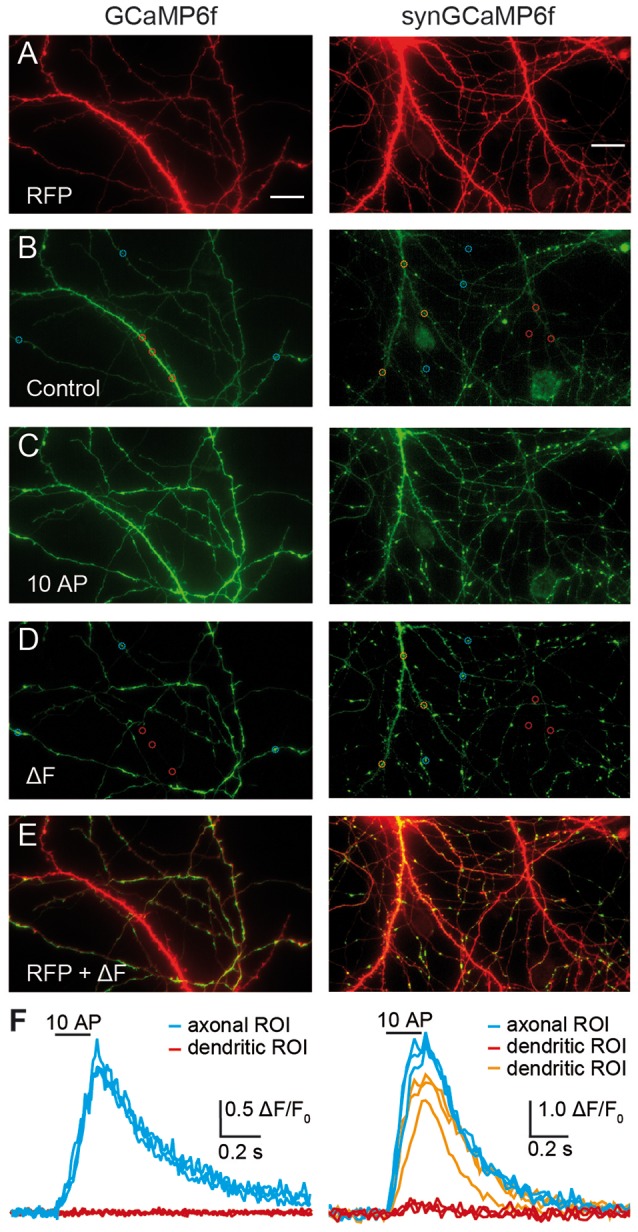
Targeting of GCaMP6f to synaptic terminals improves signal intensity for presynaptic Ca^2+^ transients. Cultured hippocampal neurons were co-transfected with RFP and GCaMP6f (left panels) or RFP and synGCaMP6f (right panels). **(A)** The RFP-fluorescence (red) shows axons and dendrites of a transfected neuron, of which the soma is positioned outside of the shown area due to its intense fluorescence. **(B)** The green fluorescence of GCaMP6f (left) is more homogeneously distributed along all processes and is stronger in thicker dendrites. In contrast, synGCaMP6f emphasizes punctate, presumably synaptic areas and is less intense over axonal shafts and dendrites. Circles depict putative presynaptic boutons (blue) and dendritic sample regions of interest (ROIs; red or orange). **(C)** A train of 10 action potential (AP) at 50 Hz increased fluorescence levels. Images in **(B,C)** are documented with identical light and exposure conditions. **(D)** Subtraction (ΔF) of control fluorescence **(B)** from the signal during stimulation **(C)** isolates the cellular compartments with enhanced fluorescence by stimulation. Circles show same ROIs as in **(B)**. **(E)** Overlay of subtraction **(D)** and RFP **(A)** images identifies the preferential position of active compartments on thinner axonal branches. The intensely labeled left dendrite (identified by its numerous spines) in the synGCaMP6f-picture shows green fluorescence in its upper parts. **(F)** Quantitative evaluation of the fluorescence intensity changes of the axonal/presynaptic traces (blue) of the ROIs shown in **(B,D)** during stimulation. ROIs from dendritic compartments (red traces, red ROIs shown in **B,D**) show no change, as postsynaptic receptors are pharmacologically blocked or, in the ROIs of more distal branches (orange traces, orange ROIs shown in **B,D**) have enhanced fluorescence, possibly due to backpropagating APs. Scale bar in **(A)** for all panels: 20 μm.

Data analysis of imaging recordings of Ca^2+^ transients was done with FIJI/ImageJ (National Institute of Health, MA, USA) and IgorPro (Wavemetrics, Lake Oswego, Oregon). Up to 50 regions of interest (ROIs) per measurement area were drawn around active boutons as indicated by stimulation with a train of three AP. Active boutons were identified in a picture isolating regions that showed an increase in fluorescence (ΔF; Figure 1) by subtracting the averaged picture of frame numbers 10–20 (control before stimulation) from the average of 11 consecutive frames around the maximal response. On this picture, ROIs were placed on active boutons by use of the plugin “Time Series Analyzer V3” with an AutoROI diameter of 10 pixels. To quantify fluorescence changes in individual boutons, we first applied the commonly used (Iwabuchi et al., 2014) “Subtract Background…” tool of ImageJ (employing a “rolling ball” algorithm with a radius of 20 pixels ≈ 10 μm), to remove the background signal deriving from faint autofluorescence and the dark current of the camera. For each ROI and each frame, the mean of the four highest fluorescence pixels was calculated by use of a self-made macro. The area of four pixels (0.85 μm^2^) corresponds to the size of a normal bouton and the restriction to the four brightest pixels avoids the problem of the relevance of the ROI size in relation to the area of increased fluorescence. Further calculations used IgorPro to average for each ROI the value of frame 10–20 as a background control (F_0_). Changes were calculated as the change of fluorescence intensity (F–F_0_ = ΔF) divided by the control (ΔF/F_0_) for each ROI. Single AP responses were analyzed after averaging four consecutive recordings already within ImageJ, and for analysis of the individual amplitudes the traces were binomial Gaussian smoothed (IgorPro) to improve the signal-to-noise ratio. In 100 Hz recordings (10 ms sampling) we used coefficient 5 (employing five passes of binomial smoothing), in 50 Hz recordings (20 ms sampling) coefficient 3.

### Statistical Analysis

No statistical methods were engaged to predetermine sample size, instead, we based our experimental design on numbers reported in previous studies. The experiments were not randomized, and investigators were only partially blinded during experiments and analyses. Statistical tests were performed with Prism (GraphPad Software, La Jolla, CA, USA). If samples met the criteria for normality, we used a Student’s *t*-test to compare two groups. Data are presented as means ± SEM. Significance levels were as indicated in figures: *P < 0.05; **P < 0.01 and ***P < 0.001.

## Results

The study of presynaptic calcium signaling using hippocampal neurons in culture has the advantage that almost all synapses are in a horizontal plane. This allows superior visibility and the observation of changes of fluorescence in many individual boutons in parallel. To measure Ca^2+^ transients in identified transfected neurons, we co-transfected the calcium-indicator protein GCaMP6f with RFP, allowing to search for positive neurons on the RFP channel (excitation at 640 ± 20 nm). Healthy transfected neurons showed strong fluorescence in the soma (not shown), several intensely labeled dendrites mostly with spines, and a fine arbor of thin axonal processes (Figure 1A). Almost all of these cells were also positive for the green fluorescence of GCaMP6f or synGCaMP6f, a variant fused to the synaptic vesicle protein synaptophysin, and visible on the green channel (excitation at 470 ± 20 nm). GCaMP6f-expressing neurons showed green fluorescence throughout the cytoplasm, leading to brighter labeling of soma and dendrites compared to axons (Figure 1B, left). SynGCaMP6f expression revealed a more punctate pattern of the fluorescence that was enriched at synaptic boutons, whereas labeling of dendrites and somata appeared more moderate (Figure 1B, right). To identify responsive regions of boutons, short bursts (3–10 stimuli) of APs were applied. Each stimulation increased the fluorescence intensity (Figure 1C). We visualized this by subtracting a control frame before stimulation (like Figure 1B) from a frame during stimulation (like Figure 1C) leading to a ΔF image (Figure 1D). Particularly axonal regions showed punctate-like putative presynaptic boutons (sample regions in blue circles, Figures 1B,D). In contrast, dendritic regions (samples in red or orange circles, Figures 1B,D) responded less frequently. Evaluation of the changes in fluorescence intensity indicated a clear increase of calcium in the axon-associated putative presynaptic boutons (Figure 1F, blue traces) but only in some dendritic areas (Figure 1F, orange traces). Other dendritic regions showed almost no Ca^2+^ transients (Figure 1F, red traces).

**Figure 2:**
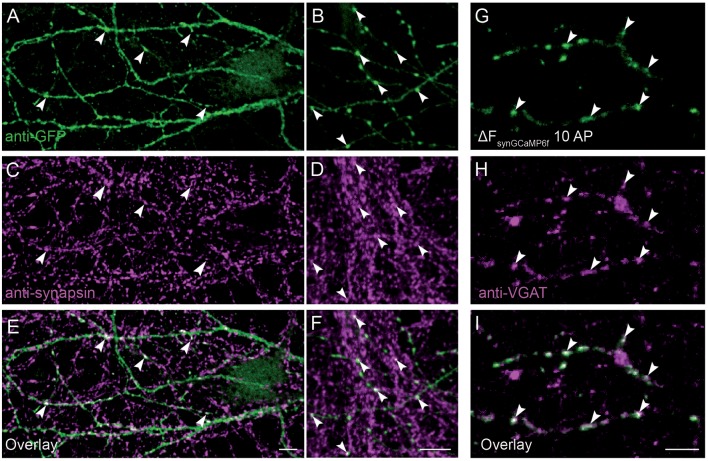
Presynaptic localization of synGCaMP6f. **(A,B)** Anti-green fluorescent protein (GFP) antibodies were used to enhance the synGCaMP6f signal (green) and showed cell processes with various varicosities, presumably mostly at presynaptic boutons. **(C,D)** To verify this, we performed double labeling with anti-synapsin1 (magenta). **(E,F)** Synaptic GFP-positive varicosities were also immunopositive for synapsin1 (white, arrowheads), indicating the enrichment of synGCaMP6f in presynaptic boutons. **(G)** The subtraction figure (ΔF, like in Figure 1D) indicates regions with synGCaMP6f fluorescence increase during stimulation with 10 AP. **(H)** GABAergic presynaptic boutons are identified by incubation with an antibody against VGAT::oyster prior to calcium imaging. **(I)** The overlay (white) identifies the localization of the synGCaMP6f fluorescence transients (green) to the VGAT-positive GABAergic presynaptic compartments (magenta). Note that no all GABAergic presynapses in this area belong to the neuron that was transfected with synGCaMP6f, as numerous VGAT-positive boutons show no synGCaMP6f signal. Scale bars in **(E,F,I)**: 10 μm.

Co-localization of synGCaMP6f with presynaptic markers was examined by two different methods. On the one hand, we performed double staining of synapsin1 and synGCaMP6f (Figures 2A–F). Since stimulation was missing, we enhanced the synGCaMP6f signal by anti-GFP labeling (Figures 2A,B), which showed various varicosities similar to those obtained under stimulation (Figure 2G). Most of those tentative synapses were also immunoreactive for synapsin1 (Figures 2C–F, arrowheads), indicating that synGCaMP6f is indeed enriched in presynaptic boutons. In another set of experiments (Figures 2G–I) the neurons were incubated with an antibody against a VGAT prior to measurements of calcium dynamics to label presynaptic boutons of GABAergic neurons (Figure 2H). In synGCaMP6f-transfected inhibitory neurons the regions of stimulus-induced fluorescence increase that were isolated by subtraction (Figure 2G) clearly co-localized with VGAT (Figure 2I), indicating the presynaptic boutons as the locus of synGCaMP6f-measured calcium transients.

Due to the more focused fluorescence of synGCaMP6f enriched in presynaptic boutons, we selected this presynaptic GECI for further analysis of presynaptic Ca^2+^ transients.

### synGCaMP6f Is a Fast and Reliable GECI

**Figure 3:**
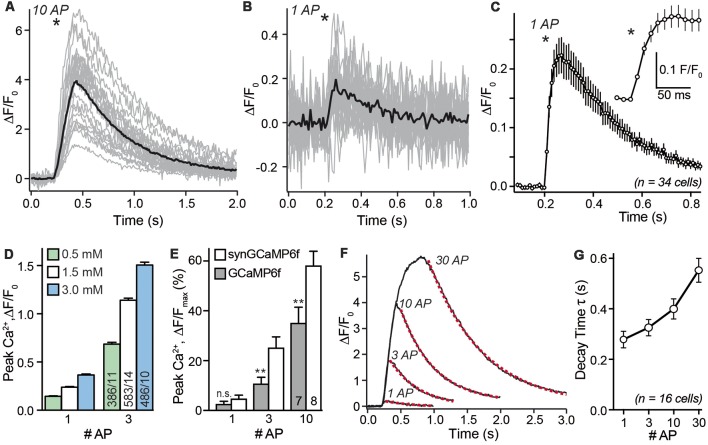
Stimulus-response relationship of synGCaMP6f-measured Ca^2+^ transients. **(A)** Stimulation with a train of 10 AP (50 Hz) reliably induced a transient increase in synGCaMP6f fluorescence that varied in size. The sample recording evaluated 23 ROIs of presynaptic boutons from a single neuron (gray lines). Black line shows averaged response; asterisk indicates onset of the stimulation. Panel **(B)** in the same ROIs shown in **(A)** a single AP elicited smaller calcium transients. Black line, averaged response to one AP. **(C)** Combining the averaged responses of 34 neurons, representing 887 ROIs, reveals the kinetics of fluorescence changes in response to a single AP. The circles illustrate the fluorescence of a 10 ms exposure period (recording with 100 Hz) averaged from 34 separate recordings. The maximum of the synGCaMP6f fluorescence intensity is sampled 60–70 ms after the AP and it lasts more than 0.8 s to reach the baseline level again. Inset shows the initial response at higher resolution. Asterisks indicates onset of stimulation. **(D)** Recording of presynaptic calcium transients induced by one or three APs in different concentrations of extracellular calcium [(Ca^2+^)_e_]. In normal recordings, 1.5 mM [(Ca^2+^)_e_] was used. **(E)** Peak values of Ca^2+^ transients in response to increasing numbers of APs are compared between GCaMP6f and synGCaMP6f and normalized to F_max_ as revealed with ionomycin application at the end of each experiment. These measurements indicate stronger fluorescence changes with the synaptophysin-coupled GCaMP. **(F)** Averaged responses (compare black lines in **A,B**) from 32 presynaptic boutons of a single neuron to 1, 3, 10 and 30 APs that underwent a mono-exponential decay time fit (dotted red line). **(G)** Analysis of decay time in >500 presynaptic ROIs from 16 neurons reveals a moderate increase to longer AP trains. Data in **(C–E,G)** are mean ± SEM; in **(E)**, columns with equal stimulation are compared by student’s *t*-test; n.s.: *p* > 0.05; **p < 0.01.

After choosing a recording area within the axonal region of a single transfected neuron we investigated the presynaptic calcium transients in response to single APs and small bursts. As shown in a representative recording in Figure 3A, the fluorescence changes of 38 individual synaptic boutons from a single neuron were recorded in parallel (gray traces) but varied strongly. The average synGCaMP6f fluorescence of 887 boutons from 34 hippocampal neurons increased more than four-fold compared to the resting fluorescence (ΔF/F_0_: 3.51 ± 0.06, *n* = 887/34) upon stimulation with a train of 10 stimuli (50 Hz). This increase was fast and continued during the stimulation period (Figure 3A). Under stimulation with three AP, the same presynaptic boutons more than doubled their fluorescence (ΔF/F_0_: 1.22 ± 0.05; *n* = 887 boutons/34 neurons). Importantly, even stimulation with single APs induced detectable signals in individual boutons (Figure 3B) that averaged to 25.8 ± 0.1% fluorescence increase (ΔF/F_0_: 0.258 ± 0.001; *n* = 34/887; Figures 3C,D). Longer trains of 30 AP induced a calcium transient that increased the fluorescence almost six-fold (ΔF/F_0_: 5.36 ± 0.07; *n* = 877/34). The response to this 30 AP train showed a steep increase in the beginning but a weaker rise in the later phase of the stimulation period. However, only few boutons reached a constant maximum as would be expected for a full saturation of the recording dye. Since stimulation with longer stimulus trains (30 AP) showed varying grades of saturation (Figure 3F), we focused further experiments on single AP and shorter trains. Repeated stimulation with 10 AP bursts showed less than 5% run-down of the response after three stimulations (not shown). When the neurons were kept in bath solutions with different extracellular calcium concentrations [(Ca^2+^)_e_], the response to 1 AP was reduced to 0.144 ± 0.005 in 0.5 mM [Ca^2+^]_e_ and enhanced to 0.365 ± 0.011 in 3 mM [Ca^2+^]_e_ (Figure 3D). Likewise, the response to three AP was smaller (0.686 ± 0.018) with 0.5 mM [Ca^2+^]_e_ and larger (1.51 ± 0.03) in 3 mM [Ca^2+^]_e_.

In a subset of experiments, we compared the fluorescence change between recordings using GCaMP6f or synGCaMP6f. We normalized it to the maximal value (F_max_) after we equilibrated the intra- and extracellular Ca^2+^ concentration using ionomycin. In this recordings a single AP induced an increase of 2.7 ± 1.0% (GCaMP6f; *n* = 128/7) or 4.9 ± 1.3% (synGCaMP6f; *n* = 260/8) of the F_max_. For GCaMP6f or synGCaMP6f three AP induced 10.9 ± 2.5% or 25.3 ± 4.2% and 10 AP increased the fluorescence by 35.3 ± 6.2% or 58.3 ± 5.6% of F_max_ (Figure 3E), respectively.

As expected from a GECI, there are also well-known limitations for the use of synGCaMP6f in assessing rise or decay times of the presynaptic Ca^2+^ signal. Averaging the mean response of all boutons of a neuron (black line in Figure 3B) for all neurons recorded with 100 Hz recording frequency reveals a time-to-peak in fluorescence change of 60–70 ms, and a 10%–90% rise time of less than 40 ms (Figure 3C). This value is clearly much slower than the time-to-peak in actual presynaptic Ca^2+^ current recordings (Borst et al., 1995), and slower than values recorded with other calcium indicators such as Fluo5F (Liu et al., 2014; Brockhaus et al., 2018). Similarly, the decay time of the fluorescence signal of synGCaMP6f in response to a single AP is slow with 279 ± 33 ms and increased only moderately when the amount of stimulation is enhanced (Figures 3F,G). These data indicate that the kinetics of the calcium transients as recorded with synGCaMP6f are dominated by intrinsic properties of the indicator, for example, its buffering properties. On the other hand, the slow kinetics allows longer light sampling (20 ms) and a related recording frequency of 50 Hz without loss of the maximal response, which improves the signal-to-noise ratio.

**Figure 4:**
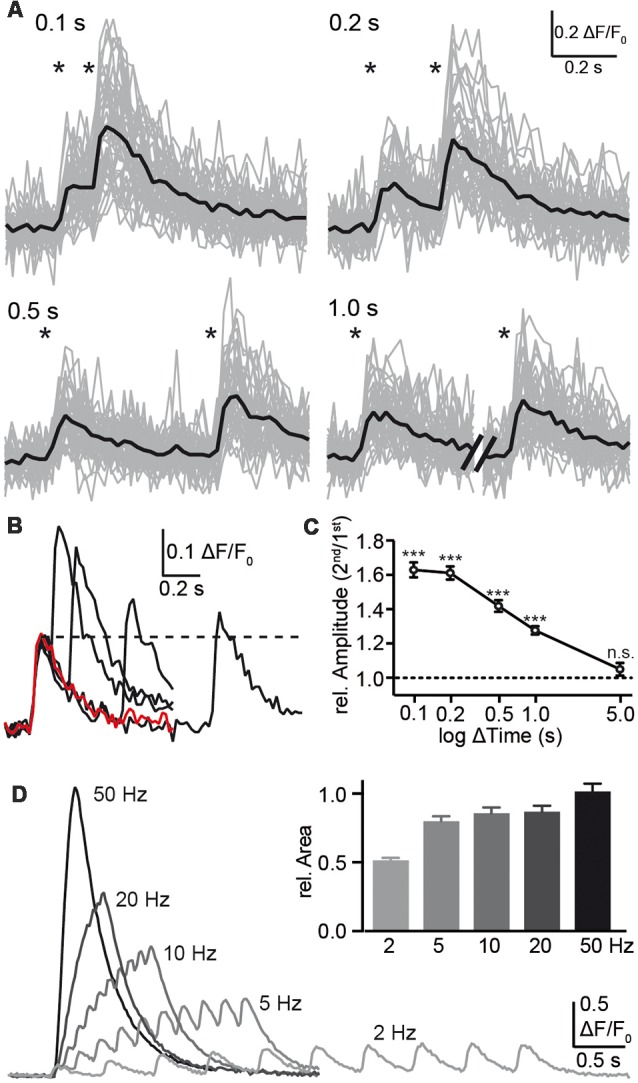
Analysis of paired-pulse facilitation (PPF) using synGCaMP6f. **(A)** Pairs of single AP stimulations with intervals between 0.1 and 1.0 s as indicated by asterisks induced an enhancement amplitude in Ca^2+^ transients following the second AP. Sample recordings show the strongest enhancement with the shortest possible interval at 0.1 s. Black traces, averaged responses from 47 ROIs. **(B)** The overlay of the averaged responses (black traces from the recordings in **(A)** are compared to the response to a single AP (red trace) of the same neuron. The dotted line indicates the amplitude maximum of the first calcium transient for comparison. **(C)** Summary of presynaptic paired-pulse ratios determined from calcium transients of 29 neurons shows a long-lasting facilitation of the presynaptic calcium response with significant amplification even at 1 s interval. For the shorter intervals <1 s, the response to a single stimulus (red trace in **B**) was subtracted prior to the evaluation due to ongoing decay of the first transient. Data in **(C)** are mean ± SEM of 29 neurons. Significance was tested in **(C)** by a paired student’s *t*-test comparing ΔF/F_0_ values of the first and second response; significance levels are indicated with ***p < 0.001, n.s.: *p* > 0.05. **(D)** Trains with 10 AP were applied with different frequencies as indicated and the area under the curves were normalized to the response to the standard frequency 50 Hz (inset; *n* = 148/3).

Despite the limitations with respect to kinetics, the observation that individual presynaptic calcium transients respond reliably and robustly to single APs opens the possibility to test presynaptic short-term plasticity, at least for time intervals of 100 ms or more. Consistently, a second stimulation within 1 s or shorter elicited a larger amplitude in presynaptic calcium compared to the initial transient (Figure 4), indicating presynaptic paired-pulse facilitation (PPF). The observation of calcium transients in presynaptic boutons offered a focus on the first steps of synaptic transmission and a facilitation already prior to transmitter release. The paired-pulse ratio was 1.63 ± 0.04 (*n* = 29 cells) with a stimulus interval of 100 ms, decreased to 1.28 ± 0.02 (*n* = 29) with 1 s interval and was not detectable after 5 s (Figure 4C). For shorter stimulus intervals, the response to a single stimulus (red trace in Figure 4B) was subtracted prior to the evaluation to make sure that the second amplitude is not overestimated. Due to the slow increase of the response to single APs with more than 50 ms time-to-peak (Figure 3C) and a recording frequency of 50 Hz (20 ms exposure time), paired-pulse intervals shorter than 100 ms were not used. We measured the PPF in the dendritic compartment and found it significantly smaller than the PPF in the axonal boutons. This increases our confidence that the bouton signal was distinct.

Additionally, we tested the importance of the stimulation frequency and applied a 10 AP burst with different frequencies (2–50 Hz; Figure 4D). Faster stimulus series induced larger fluorescence changes but this may result mainly from the summation of responses to individual stimuli together with enhanced responses due to PPF (see above). Evaluation of the area under the curve, which corresponds to the amount of increasing calcium, was not strongly affected by frequencies of 5–50 Hz (Figure 4D, inset).

### Analysis of Ca^2+^ Channel Subtypes With synGCaMP6f

**Figure 5:**
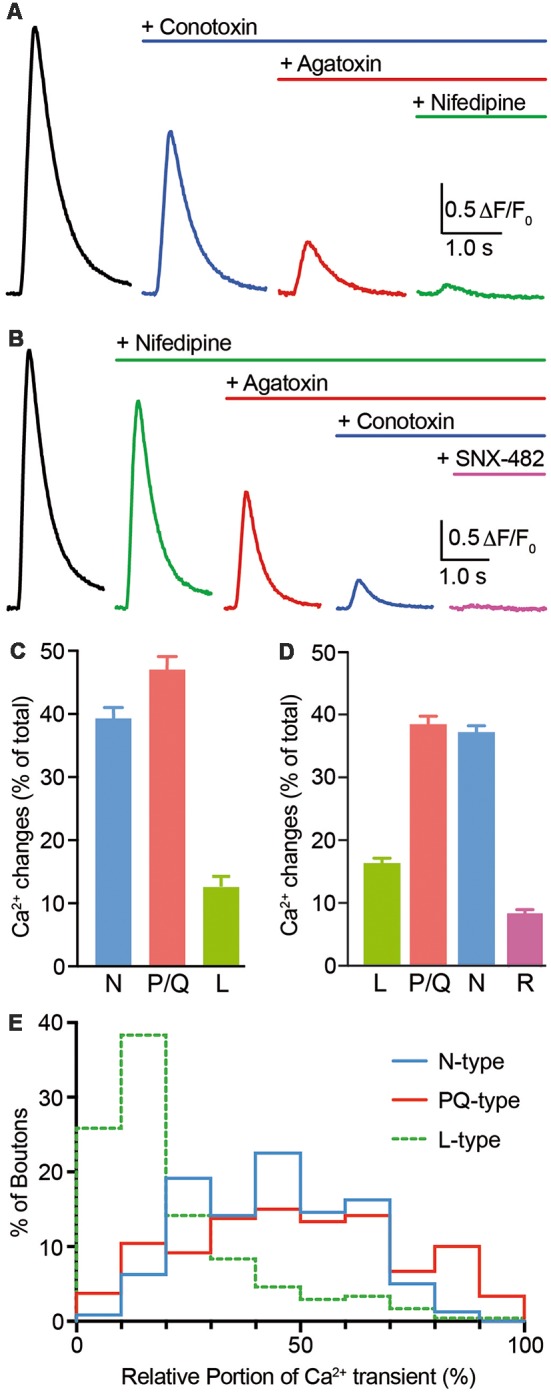
Voltage-gated calcium channel (VGCC) subtype contribution to Ca^2+^ transients monitored by synGCaMP6f. **(A)** Consecutive application of ω-conotoxin GVIA (2 μM; N-type Ca^2+^ channel blocker), ω-agatoxin IVa (0.4 μM; P/Q-type Ca^2+^ channel blocker) and nifedipine (20 μM; L-type Ca^2+^ channel blocker) was employed to evaluate the contribution of different VGCC subtypes to fluorescence changes induced by trains of 10 APs (50 Hz). Color-coded traces show averaged responses from 239 ROIs in nine neurons before (black) and after sequential addition of blockers as indicated. **(B)** Independent set of experiments (*n* = 235/11) similar to **(A)** but with altered order of application and addition of SNX-482 (0.5 μM; R-type Ca^2+^ channel blocker). **(C,D)** Bar diagrams summarizing the relative contribution of Ca^2+^ channel subtypes from experiments in **(A,B)**. Both diagrams confirm that N- and P/Q-type VGCCs contribute mostly and almost equally to presynaptic calcium transient in hippocampal neurons. **(E)** Histogram presenting the wide variability of VGCC subtype contributions to Ca^2+^ transients at the level of individual presynaptic boutons (*n* = 239/9) as evaluated from the recordings in **(A,C)**. A few percent of the boutons even had more than 50% nifedipine-sensitive L-type Ca^2+^ current.

Presynaptic calcium rise is dominated by N- and P/Q-type Ca^2+^ channels (Ca_V_2.1 and Ca_V_2.2, respectively; Li et al., 2007; Cao and Tsien, 2010). These channels can be blocked by the highly specific toxins ω-conotoxin GVIA (conotoxin; N-type blocker) and ω-agatoxin IVa (agatoxin, P/Q-type blocker). We, therefore, investigated if synGCaMP6f is suitable to determine the contribution of different Ca^2+^ channel subtypes to presynaptic calcium transients. We applied the blockers in concentrations that were successfully used by others to achieve full channel-specific blockade (Randall and Tsien, 1995; Li et al., 2007; Cao and Tsien, 2010). First, we stimulated with trains of 10 AP to get a robust signal-noise ratio even after reduced responses due to a partial blockade. Consecutive use of conotoxin, agatoxin and the L-type channel (Ca_V_1.x) blocker nifedipine (*n* = 239/9; Figures 5A,C) revealed a predominant role of P/Q-type (47.4 ± 1.7%) and N-type (39.7 ± 1.3%) Ca^2+^ channels and a minor contribution from L-type (12.9 ± 1.3%). To overcome bias imposed by the order of blocker application (Cao and Tsien, 2010), we performed a second set of recordings with sequential application of nifedipine, agatoxin and conotoxin (Figures 5B,D). In addition, we applied the R-type antagonist SNX-482 (*n* = 235/11) to identify the source of the small transient sometimes left in the first set of recordings. Again, the majority of calcium influx results from P/Q-type (38.4 ± 1.5%) and N-type (36.9 ± 1.3%) channels. However, in this order 16.5 ± 1.1% of the calcium influx was due to L-type channels and an additional 8.2 ± 0.9% were shown to be sensitive to the R-type antagonist SNX-482.

By fluorescence measurements of many individual presynaptic boutons with Ca^2+^ channel blockers, synGCaMP6f allowed the analysis of the distribution of the different Ca^2+^ channel subtypes in individual boutons. This evaluation was done on the first set of recordings with 10 APs to focus on the comparison of N- and P/Q-type channels. We asked whether both subtypes were equally strong in all boutons or showed individual divergence. We calculated the relative contribution of N-, P/Q- and L-type channels for the calcium transient of each bouton. A frequency histogram shows the frequency of the relative contribution of each channel subtype (Figure 5E). For N- and P/Q-type channels, contribution ranges from almost equal to boutons that are dominated by either one of the two major subtypes. L-type channels contributed only little to the calcium transients in most boutons (<20%; green line in Figure 5E) but dominated the response in few boutons with up to 70% of the total calcium. This diversity among individual presynaptic boutons indicates an independent distribution of Ca^2+^ channel subtypes. Together with the widespread amplitude distribution in response to single stimuli or short bursts, our results suggest that data from individual boutons can be treated as independent in the statistical evaluation of such experiments.

## Discussion

With the invention of GCaMP as a genetically encoded fluorescent calcium indicator (Nakai et al., 2001; Chen et al., 2013), a potent tool for the observation of calcium dynamics has become available. Originally intended as an indicator for active neurons (Chen et al., 2013), it also allows the investigation of changes in the internal calcium concentrations in subcellular compartments, including small regions such as individual presynaptic boutons (Reese and Kavalali, 2016; Brockhaus et al., 2018). We focussed on the presynaptic compartments of standard preparations like cultured hippocampal neurons that typically have diameters of 1 μm or smaller. To avoid a mixture with signals from adjacent postsynaptic areas we used three independent methods: (1) we coupled GCaMP6f to the presynaptic protein synaptophysin to localize the calcium indicator by intrinsic sorting mechanisms preferentially to presynaptic loci; (2) we pharmacologically blocked ionotropic glutamate and GABA receptors with CNQX, AP5 and bicuculline to prevent postsynaptic signaling and subsequent returning network activity; and (3) we sparsely transfected only a minority of neurons to exclude co-labeling of pre- and postsynaptic areas. Additionally, co-labeling with presynaptic markers, like the vesicular GABA-transporter VGAT or synapsin, provided support for the presynaptic localization of synGCaMP6f fluorescence transients.

Evaluation of putative dendritic areas of the transfected neurons without labeling in the presynaptic boutons (Figure 1) only rarely showed transients, even in cells transfected with the uncoupled GCaMP6f. SynGCaMP6f showed a more punctual fluorescence, often weak without stimulation. The low baseline fluorescence may in part be due to the small volume of presynaptic boutons but also reflects a low resting concentration of free calcium as typical for healthy physiological conditions of these neurons.

We used fluorescence changes of synCGaMP6f as a measure for changes in the internal concentration of free Ca^2+^. The baseline fluorescence F_0_, recorded during the resting state before stimulation, is the central reference point for the quantification of the relative changes (F–F_0_)/F_0_. In neurons, in which the intracellular Ca^2+^ concentration is important for many signaling cascades, the resting calcium concentration is strongly controlled. Still, the use of only this baseline fluorescence would be a disadvantageous aspect of the evaluation. To make the resting calcium concentration a relevant measure as a reference point, it is a prerequisite that the neuron was not spontaneous active in the seconds before the recording. Since we used blockade of fast glutamatergic transmission, we hardly saw spontaneous activity in the neuronal network in long lasting observation periods (data not shown) and can exclude a disturbance of our evaluations. Additionally, spontaneous activity or recovery would be seen in the control period of the recording before stimulation (duration 0.2–0.4 s) and exclude this measurement from further use.

Opposing the baseline fluorescence, the maximal fluorescence can serve as a second reference point for the quantification. It can be seen during application of an ionophore (e.g., ionomycin, Hoppa et al., 2012) or long lasting stimulation (e.g., 100 AP, 100 Hz; Ermolyuk et al., 2012). Here, we used this method to compare the fluorescence changes measured with GCaMP6f and synGCaMP6f, a form targeted to the presynaptic vesicles by coupling to synaptophysin. Short bursts induced significantly stronger responses with synGCaMP6f. This may result from a position closer to the presynaptic calcium channels which would result in higher Ca^2+^ transients in its surrounding area as the calcium gradient is steep during APs near the presynaptic membrane (Stanley, 2016). One may speculate that the higher response of the targeted synGCaMP6f does not result from a change in the sensitivity but from its more relevant position around synaptic vesicles near to the presynaptic Ca^2+^ channels. Overall, the ionophore application makes recording much more sophisticated and time consuming and does not lead to more powerful results.

How much change in fluorescence can we see during neuronal activity? Inducing a short burst by a stimulation of three APs already doubled the fluorescence (ΔF/F_0_ > 1) and was immediately visible. Stronger bursts (10–30 AP) increased the fluorescence several fold. Even the presynaptic calcium transients during a single AP could be recorded. With reasonable signal-noise ratio after averaging four consecutive single AP stimulations with 10 s distance, fluorescence increased on average by 25.8% (ΔF/F_0_ = 0.258). This presynaptic response to single APs is slightly higher than the transients described in the original study inventing GCaMP6f (Chen et al., 2013; total cell 19%). This may be explained with different conditions when evaluating somatic fluorescence, e.g., the density of calcium channels in relation to the volume is lower.

Although hippocampal neurons are shown to have backpropagating APs, we only saw substantial Ca^2+^ transients in some dendritic compartments (Johnston et al., 1996; Waters et al., 2005). The efficacy of backpropagating APs to induce Ca^2+^ transients depends on different parameters like the rather low density of Ca^2+^ channels in relation to the higher volume of the dendrite. Also, the membrane potential affects the highly present A-type K^+^ channels and results in a shunt of depolarization decreasing the amplitude to values ineffective in activating Ca^2+^ channels (Hoffman et al., 1997; Waters et al., 2005). The delicate regulation of AP backpropagation may even lead to a dichotomy within the same neuron (Golding et al., 2001; Sheffield and Dombeck, 2015). Thus, the increase in synGCaMP6f fluorescence may be below the threshold for visibility in dendritic compartments when compared to presynaptic Ca^2+^ transients, where the synaptophysin-coupled indicator is enriched. This does not exclude a backpropagating component of the stimulation-induced AP.

The kinetic of the presynaptic response was rather slow, taking 60–70 ms to reach the peak. This is in line with the original description of GCaMP6f (80 ms; Chen et al., 2013) but slower than seen with other dyes like fura-2 or mag-fura-5 (Atluri and Regehr, 1996), Fluo5F (Liu et al., 2014; Brockhaus et al., 2018) or OGB (Kirischuk et al., 1999). Comparatively, electrophysiological recording of the Ca^2+^ current, that induces the rise in internal free Ca^2+^ concentration lasts about 1 ms (Augustine et al., 1991; Kawaguchi and Sakaba, 2015).

Thus, the rise time in GCaMP6f recording does not describe the kinetics of free presynaptic calcium changes but rather indicates binding constants of calcium ions to the calmodulin part of the indicator. Also, the decay time constant of almost 280 ms for single AP transients is substantially slower than seen with other methods, but in the ranges described earlier (Chen et al., 2013). In conclusion, recording of calcium transients with (syn-)GCaMP6f does not allow investigating the kinetics of fast changes in Ca^2+^ concentration. On the other hand, the slow kinetics allow exposure times (as long as 20 ms) and low recording frequency (resulting in 50 Hz), which improves the signal-noise ratio and helps with the evaluation of small regions like presynaptic boutons of hippocampal or cortical cells.

The amplitude of the calcium transient in response to an increasing number of APs showed a steep increase in relation to the AP number. However, the response to three AP strongly exceeded the three-fold increase of a single response (1.22 > 3 × 0.258). This led to the question of whether synGCaMP6f enabled us to measure presynaptic short-term plasticity like PPF within closely timed APs in cultured hippocampal neurons. To further investigate this, stimulus pairs were applied with different time intervals between 0.1 and 5 s. Shorter time intervals were not possible as a clear maximal response to the first pulse has to be established before the second stimulation which was only reached after 60 ms or more, probably due to intrinsic features of the sensor protein (see above). The stimulus pairs of up to 1 s distance induced significant presynaptic PPF and reached more than a doubling with 0.1 s pairs. Presynaptic origin of the usually postsynaptically recorded PPF is a well-known phenomenon (Zucker and Regehr, 2002; Jackman and Regehr, 2017) with several possible causes. It may account from enhanced calcium entry, reduced buffering or increased probability of vesicle release due to the recruitment of additional synaptotagmin subtypes (SyT7, Jackman et al., 2016). Here we employ a method directly to observe modifications of presynaptic Ca^2+^ concentrations during paired-pulse stimulation. One possible mechanism for the observed presynaptic PPF of Ca^2+^ transients is buffer saturation and subsequently an enhanced transient of free Ca^2+^ in response to the second pulse. Also, a stronger calcium current itself induced by the second pulse due to binding of the calcium channels C-terminus (EF-hand-like motif of Cav2.1; Chaudhuri et al., 2004) to calcium-bound calmodulin and other intrinsic Ca^2+^ buffers is known. Both mechanisms may be enhanced by the transfected GECI, synGCaMP6f, as it uses a calmodulin domain for calcium binding (and sensing) and adds additional calcium buffer capacity to the neuron (McMahon and Jackson, 2018). This needs to be considered in direct comparison of results with and without GCaMP but is a problem of almost all fluorescent calcium indicators. In a study of different conditions that were all measured with GCaMP, the interference is equal and hardly perturbs the result. In a recent study on hippocampal PPF, Jackman et al. (2016) used bulk-loading of CA3 fibers with different calcium indicators in brain slices and stimulation in presence of NBQX and picrotoxin to suppress postsynaptic responses. They saw no presynaptic PPF in CA3-CA1 synapses in photodiode recordings. Observation of calcium dynamics in individual presynaptic boutons, as presented here, showed PPF. This discrepancy may result from the different recording techniques and a broader mixture of synapses in cell culture of hippocampal neurons, from different conditions in brain slices vs. cell culture or from different intracellular calcium buffering, as this is a crucial factor for presynaptic PPF (Jackman and Regehr, 2017).

Another set of experiments scrutinized the contribution of different calcium channel subtypes to the presynaptic calcium transients in response to a burst of APs in hippocampal neurons. We are aware that the quantification depends on the assumption of an almost linear relation of calcium concentration and fluorescence change. Two strategies were used to minimize related constraints: With 10 AP, a stimulus was chosen that induced a response of less than 60% of the saturated indicator (Figure 3E). Additionally, two different orders of blocker application avoided corruption by non-linear fluorescence-calcium relation, e.g., if higher fluorescence showed weaker changes with related changes in calcium concentration. As expected, the transients were dominated by P/Q- and N-type channels, which almost equally contributed to the response. Additionally, nifedipine-sensitive L-type channels had a share of more than 10% and also SNX-482-sensitive R-type channels were identified. Helton et al. (2005) found that nifedipine did not block L-type channels during stimulation with APs, but we found that nifedipine was effective. This difference may be due to the different conditions of the experiments. Cao and Tsien (2010) examined the contribution of the different subtypes in hippocampal synapses by evaluation of excitatory postsynaptic currents (EPSCs) with a comparable consecutive blocker application and also saw an almost equal share of N- and P/Q-type channels with a minor contribution of R-type channels. But with an observation of EPSCs there was no evidence for L-type channels since a block of N-, P/Q- and R-type channels completely suppressed the postsynaptic response (Li et al., 2007; Cao and Tsien, 2010). The different observation of the role of L-type channels may result from a different measure for the contribution to presynaptic calcium transients, namely the direct recording of changes in presynaptic free Ca^2+^ ions vs. recording of the final (and relevant) effect of presynaptic calcium rises, the postsynaptic current. Thus, an exclusive involvement of N- and P/Q-type channels (and a minor amount of R-type) to the release-inducing nano-domains (Eggermann et al., 2011) *via* direct coupling of calcium channels to the release machinery (Kaeser et al., 2011; Südhof, 2013) may explain that L-type-depended calcium increase is seen in the presynaptic compartments, but does not contribute to postsynaptic currents.

A special feature of the synCGaMP6f-driven presynaptic recording is the possibility to investigate large numbers of synaptic boutons individually. When evaluating the channel subtype contribution this enabled us to compare many boutons. Interestingly, the contribution of N- or P/Q-type calcium channels was widely scattered with some boutons almost completely driven by P/Q-type channels and others showing less than 10% P/Q-type contribution. A few boutons even showed more than 50% L-type dependent transients whereas most boutons had 20% or less originating for L-type channels. This wide divergence of subtype contribution is in line with the findings of Miyazaki et al. (2005) and fits to the assumption that only very few calcium channels per presynaptic bouton contribute to vesicle release (Eggermann et al., 2011; Ermolyuk et al., 2012), because a statistical distribution can induce a large variability with small numbers.

Several studies identify problems with GCaMP-transfected neurons that reach beyond the problem of enhanced calcium buffering. Different mouse lines with stable transfection of GCaMP-expression, in which the entire development was affected by the presence of the indicator, show modified neuronal activity with interictal spikes up to epileptiform events (Steinmetz et al., 2017). In AAV-infected mice moderately reduced vesicle release was described in the calyx of Held synapse more than 18 days after transfection (Singh et al., 2018) and accumulation of GCaMP6 in the nucleus occurred in the third week of transfection (Yang et al., 2018). GCaMP6m modifies the gating of Cav1.3 channels, that show enhanced voltage gated activation and calcium-dependent inactivation, which in part resembles the effect of additional apo-calmodulin, but Cav2.2 (N-type) calcium channels were not significantly affected (Yang et al., 2018). These problems have to be considered when using longer transfection periods or studying Cav1.3-dependent calcium effects but are of minor relevance in investigations on fast presynaptic transients.

Despite such drawbacks that almost all fluorescent calcium indicators show, synGCaMP6f provides many advances for the investigation of presynaptic calcium transients. What stands out is the possibility to observe the response of individual presynaptic boutons to short bursts or even single APs. In particular, the response to single APs has the advantage of not being affected by short-term plasticity, retrograde signaling or other secondary processes. This supports the comparison of data from different laboratories. Combined with the superior visibility of cultured neurons, where many synapses are positioned in one optical plane, synGCaMP6f offers a powerful tool for studies on basic presynaptic processes within large numbers of synaptic boutons and without disturbance from postsynaptic activities.

## Ethics Statement

Animal experiments were performed at the University of Münster in accordance with government regulations for animal welfare and approved by the Landesamt für Natur, Umwelt und Verbraucherschutz (LANUV, NRW, Germany), license numbers 84-02.05.20.11.209 and 84-02.04.2015.A423.

## Author Contributions

JB and MM designed the experiments. JB and BB recorded and evaluated data and prepared the figures. BB performed the immunohistochemistry. JB, BB and MM wrote and edited the manuscript.

## Conflict of Interest Statement

The authors declare that the research was conducted in the absence of any commercial or financial relationships that could be construed as a potential conflict of interest.
